# Different strategies in pointing tasks and their impact on clinical bedside tests of spatial orientation

**DOI:** 10.1007/s00415-022-11015-z

**Published:** 2022-03-08

**Authors:** J. Gerb, T. Brandt, M. Dieterich

**Affiliations:** 1 Department of Neurology, University Hospital, Ludwig-Maximilians University, Marchioninistrasse 15, 81377 Munich, Germany; 2 Graduate School of Systemic Neuroscience, Ludwig-Maximilians University, Munich, Germany; 3 German Center for Vertigo and Balance Disorders, University Hospital, Ludwig-Maximilians University, Marchioninistrasse 15, 81377 Munich, Germany; 4 Hertie Senior Professor for Clinical Neuroscience, Ludwig-Maximilians University, Munich, Germany; 5 Munich Cluster for Systems Neurology (SyNergy), Munich, Germany

**Keywords:** Spatial memory, Spatial orientation, Pointing task, Bedside test, Dementia, Vestibulopathy, Smartphone

## Abstract

Deficits in spatial memory, orientation, and navigation are often early or neglected signs of degenerative and vestibular neurological disorders. A simple and reliable bedside test of these functions would be extremely relevant for diagnostic routine. Pointing at targets in the 3D environment is a basic well-trained common sensorimotor ability that provides a suitable measure. We here describe a smartphone-based pointing device using the built-in inertial sensors for analysis of pointing performance in azimuth and polar spatial coordinates. Interpretation of the vectors measured in this way is not trivial, since the individuals tested may use at least two different strategies: first, they may perform the task in an egocentric eye-based reference system by aligning the fingertip with the target retinotopically or second, by aligning the stretched arm and the index finger with the visual line of sight in allocentric world-based coordinates similar to using a rifle. The two strategies result in considerable differences of target coordinates. A pilot test with a further developed design of the device and an app for a standardized bedside utilization in five healthy volunteers revealed an overall mean deviation of less than 5° between the measured and the true coordinates. Future investigations of neurological patients comparing their performance before and after changes in body position (chair rotation) may allow differentiation of distinct orientational deficits in peripheral (vestibulopathy) or central (hippocampal or cortical) disorders.

## Introduction

Deficits in spatial orientation can occur in both cognitive and vestibular disorders [1]. An early symptom of cognitive decline such as mild cognitive impairment or beginning Alzheimer’s dementia is a navigational impairment, which causes patients to get lost in new environments. While large deficits in spatial navigation are often noticed by the patients or their relatives, quantitative testing of the patient’s performance in orientation and navigational tasks and disclosure of preclinical deficits are difficult. Questionnaires can only give a broad estimate of one’s ability to navigate in a given environment [2]. Wayfinding studies usually require a complex setup that isn’t feasible as a clinical routine [3, 4]. However, it is desirable to include a bedside tests of spatial orientation in a neurologic assessment to uncover deficits to prevent from potentially dangerous situations [5].

One basic way in which humans or non-human primates interact with their environment is using their hands, either for direct manipulation of objects or indirect purposes, e.g., communication. Any visually triggered action or reaction towards an external stimulus requires multiple conscious and subconscious stages of cognitive processing to allow a targeted motor output. These basic activities qualify for a quick clinical bedside test of a patient’s ability to perform spatial navigation and orientation. A first prototype of our handheld pointing device using the inbuilt sensors of an Apple iPhone® showed promising results [6]. While this approach did show the principal feasibility of harnessing the sensor output from a modern smartphone for neuroscientific navigation testing, it relied on GPS and compass information, therefore, suffering from limited applicability indoors (as positioning via GPS does require an uninterrupted line-of-sight towards the satellites orbiting earth) and proneness to errors in the presence of magnetic fields.

Directed behavior with separate multimodal input and specific motor output use their respective reference systems that are only compatible after transformation processes. A first internal representation of visual input is retinotopic, based on two sets (in binocular vision) of eye-centered coordinates depending on the three-dimensional spatial position of the fovea relative to the stimulus [7, 8]. Target and effector organ placement (e.g., the right index finger and the right thumb in a visually controlled reaching task for a right-handed subject) must be integrated relative to the subject’s line of sight. This relative representation is then transformed to fit different reference systems, allowing joint-based movement plans for activation of the appropriate group of muscles required for the generation of the final output task [9]. Proprioceptive information on relative joint position and muscle contraction is used in both the initial movement plan and online correction while performing the movement to achieve a precise and adaptable finger position in grasping [10] while including online fine-tuning with binocular vision (e.g., for depth perception) [11]. This complex sensorimotor control is further influenced by higher cognitive social inter-personal aspects of interaction with the environment [12, 13] and the actual disposition of the individual, be it cultural, societal or biographical [14].

In the following, we will first discuss structural and functional aspects of spatial orientation, the specific meaning of pointing as a fundamental element of interaction with the environment and its fallibility when using pointing as a quantitative measure for spatial orientation. Then, we will analyze the shortcomings of the methods and the pointing device used in our initial description for a bedside application-based assessment [6]. Finally, we will propose a further developed pointing device with a modified standardized test paradigm and report the preliminary data of a pilot experiment in healthy participants.

## Neuroanatomical background on egocentric and allocentric spatial orientation

In trying to understand the neurocognitive backgrounds of visuomotor brain functions, Ungerleider and Mishkin in 1982 [15] first introduced the concept of two functionally and structurally separated visual systems. Based on lesion studies in rhesus monkeys, they claimed that lesions in the inferior temporal cortex produced deficits in visual object discrimination without affecting spatial orientation (by means of a landmark task), while lesions of the posterior parietal cortex produced performance deficits in spatial orientation but not in object discrimination. Goodale and Millner in 1992 [16] postulated an output-based approach of two separate and distinct visual systems with a ventral stream (also called the “what” pathway) and a dorsal stream (also called the “where” pathway) involved in processing of the localization of objects in the visual environment.

The dorsal pathway terminates in parietal and frontal movement areas and is thought to be involved in egocentric coding, preferably using egocentric coding in a gaze-centered, eye-based coordinate system [17]. fMRI recordings in humans suggest egocentric reference frames in the parietal and frontal visuo-motor areas with gaze-centered coding in the posterior parietal cortex and the dorsal premotor cortex, while body-centered reference frames seem to be used near the motor cortex, allowing body–world interaction. The ventral stream (including occipital and temporal areas involved in object recognition) is associated with allocentric, world-based coding. Lesions in the ventral stream can cause “visual form agnosia”, e.g., the inability to describe an object’s shape or color, while still being able to grasp it precisely according to its spatial and geometric properties [18]. If egocentric and allocentric cues are present, the brain is likely to integrate both and include allocentric information in the movement plan. The use of more allocentric or egocentric approaches in an individual navigation strategy likely depends on many factors, such as psychosocial elements, e.g., learned behaviour or motivation, the perceived reliability, the situational requirements (e.g., the question of speed vs. precision described as the speed–accuracy–tradeoff [19]) as well as the general mental capacity with studies showing the pronounced usage of egocentric navigation strategies in cognitive decline [20, 21]. More general, the flexibility in switching between navigational strategies is affected by subject age and overall decreases over time [22].

Relevant structures for the transformation of reference frames in visual input/motor output tasks are the superior colliculus and the frontal eye field with both directly projecting to the brainstem and to spinal cord burst generators responsible for coordination of eye and head motion [23]. The superior colliculus is a multisensory sensorimotor hub with major input from the retina and auditory and somatosensory receptors to mediate spatial orientation of gaze, ears, head and body coordinated orientation movements critical for survival in threatening situations [24–26].

The vestibular system, similar to the visual system, uses multiple reference frames. Electrophysiological studies in monkeys revealed mainly body (or world)-centred neuron tuning in the ventral intraparietal area and in the parietoinsular vestibular cortex (PIVC), whereas neurons in the dorsal medial superior temporal area were frequently close to a head-centered reference frame, showing signs of also being shifted towards an eye-based reference frame [27]. Since the vestibular organs are fixed in the petrous bone, an organ-based initial reference-frame seems likely; the multiple transformations and cross-connections towards other sensory systems are still largely unknown [28].

### Pointing, an elementary interaction with the environment

**Fig. 1:**
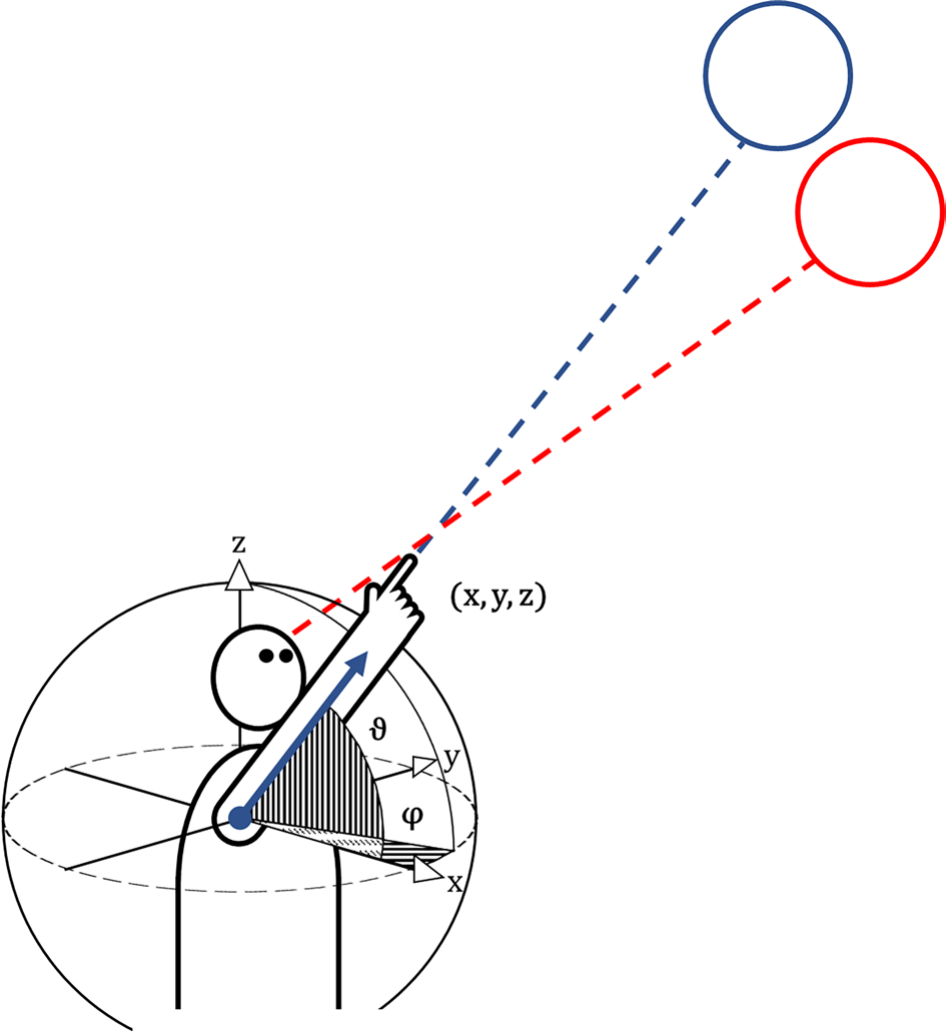
Simplified model without interlimb mobility showing the discrepancies between retinotopic pointing (red) and the corresponding pointing vector of the extended arm and index finger (blue). The pointing vector in cartesian coordinates (*x*, *y*, *z*) can be transformed into spherical coordinates (φ for Azimuth angle, ϑ for polar angle/inclination), if the origin of both coordinate systems is identical

Pointing as a common, intuitive real-life example of subject-world interaction, can meet several requirements as a social cue, a signal for raising awareness or providing directions towards an object. This action performed with eye-centered coordinates shows a systematic error if compared to an absolute, world-based coordinate system simply by the offset of the different centers of the reference systems [29]. For example, exact pointing towards an out-of-reach target such as aiming a pretend gun or in measuring angles is typically performed by closing the opposite eye thus bringing the centers of reference as close together as possible by moving the eye towards the shoulder and upper arm of the acting arm. We will use the German word “*peilen*”, a broad term for various methods of eye-based measurements or navigation with or without tools (a kind of coordinate geocaching of distance and directional angle), to describe this specialized pointing task. Both *peilen* and visually controlled grasping/reaching can be performed using transformations of the involved reference systems from eye-based to effector–organ-based and by applying online updating of movement plans. By having both input and target encoded in the same eye-coordinate based reference system, for *peilen* also a simpler solution might suffice, where the effector organs position is corrected directly based on its retinotopic visual input rather than its world-based position. This explains the use of *peilen* in, e.g., nautical navigation or military target estimation, often with the help of tools to quantify the retinotopic image. One key difference is that *peilen* requires a direct line of sight, whereas pointing can also be targeted at directions outside of the current field of view. This is achieved either by calculating an aiming vector from prior available visual information or by calculating the vector based on a mental map of the surroundings established by earlier experience. The necessary optimization of the reference system in *peilen* (where the vector of the extended arm and index finger are pointing at the target) is something humans perform as a learned automatic act from an early age. There are two strategies of real life pointing applications which serve a different purpose. In the first, the goal is not a close approximation of the extended extremity to the actual direction towards a distant target (as would be required in grasping or *peilen*), but instead the fingertip is placed exactly on the retinotopic representation of the object (similar to using the iron sights of a rifle), therefore, intersecting the line of sight. In this situation, the vector of the extended arm misses the target systematically simply by the anatomical offset between the eyes as the sensory input organ for the gaze centered visual input and the pointing extended finger of a hand as the motor effector organ, responsible for the shoulder centered reach plan (see Fig. 1).

**Fig. 2:**
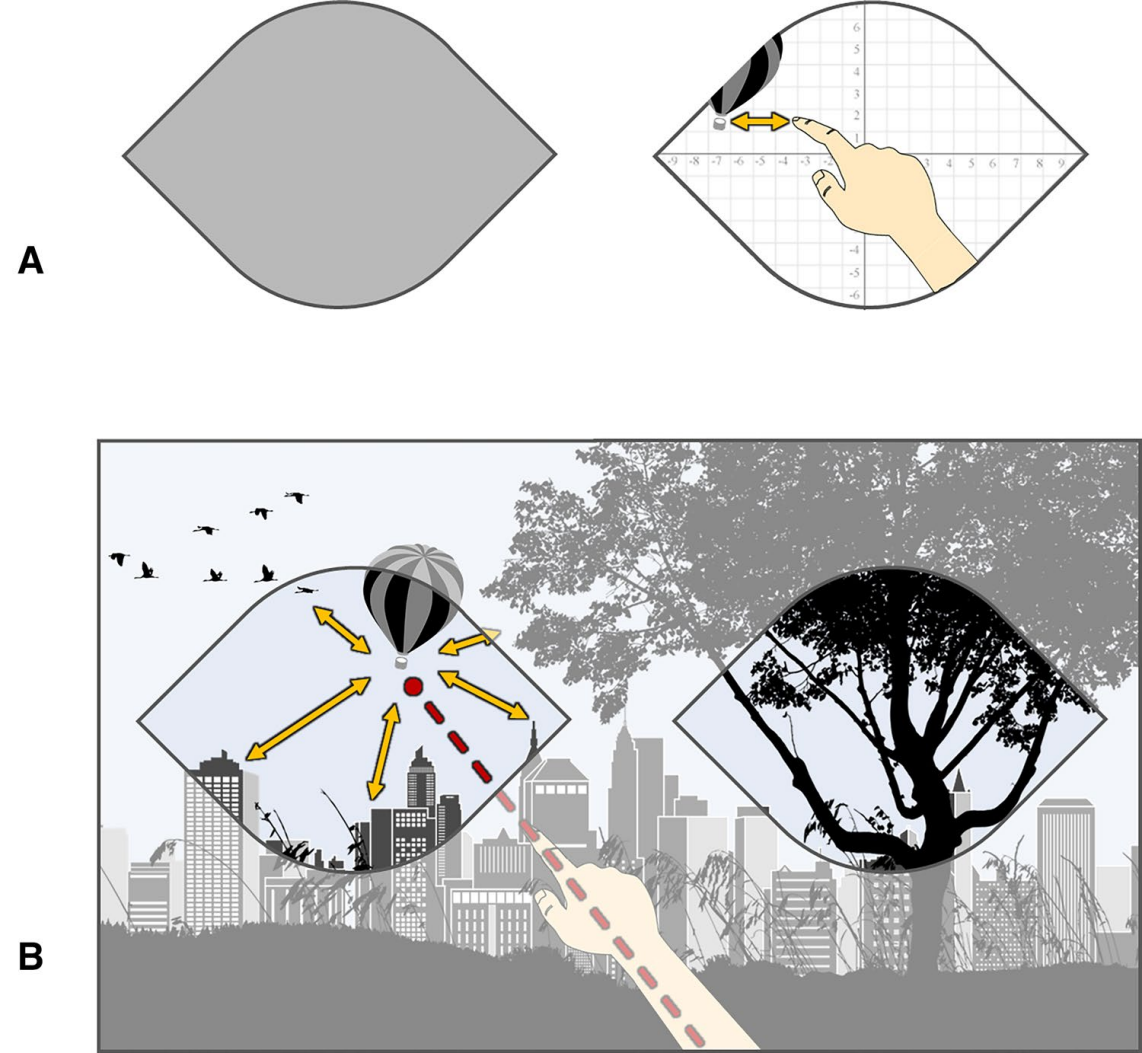
**A** Example of a retinotopic pointing strategy. The task can be performed sufficiently by adjusting the finger phalanx and the target position in a retinotopic reference frame. **B** Example of a world-based pointing strategy with the pointing vector leading towards the target in real-world coordinates. This task can only be performed by creating a mental map of the target and the interrelations with its environment

In the second strategy, the stretched arm with the index finger form a vector which is aligned to the line of sight. Since world-based and subject-based pointing strategies are usually both available when performing a pointing task, the actual strategy chosen depends on various factors. In terms of complexity, a subject space-based, retinotopic strategy only requires the visual input and an eye-centered coordinate system to encode spatial information, whereas a world-based pointing strategy requires complex transformations of the eye-centered coordinates relative to the actual 3D position of the fovea to the effector-organ centered coordinates to generate an appropriate movement plan (see Fig. 2 for details).

## Problems of an earlier approach

The previously used approach had several practical limitations [6]. As already discussed, any non-random order of targets enables the subject to simply reproduce a motor task. Selecting targets with too similar directional vectors would require a higher angular discrimination performance then the devices sensors allowed; especially when solely relying on the compass for assessing the 3D direction as an in-hospital setting usually does not allow for precise-enough GPS-based localization. As for compass information, many possible interference factors might be present in a non-standardized environment at the bedside. Even though a lot of thought was put into making the device user-friendly, the suggested use of buttons located on the device itself creates a dual task of both pointing and manually confirming the target. In sensitive enough sensors, the slight movement of pressing a button might already drift off the intended vector. Furthermore, this mode of confirmation might be too complex for elderly or cognitively impaired patients not used to handling smartphones or similar devices.

One key problem in the original design [6] is the lack of distinction between the different strategies involved when pointing. Whether a subject uses a visually controlled *peilen* approach or a world-based pointing strategy not only tests different orientation and transformation subsystems, but the measured output data is also intrinsically incompatible. By providing a laser pointer as a visualization of the pointing vector of the arm, a world-based pointing strategy was used as the calibration; a subject applying a *peilen* strategy later will, therefore, always create large deviations in their respective pointing vectors, even when performing well in a retinotopic reference frame.

Especially in an only poorly understood neuro-cognitive system, such as the representation of the outside world in an internal mental map, every measurement of tasks in this field should be as precise as possible and must, therefore, acknowledge the different aspects of retinotopic *peilen* and world-based pointing. A mix of these two, e.g., a world-based, laser-supplemented task as a calibration method and a reproduction with an unclear reference system can create large systematic errors. A poor separation of different solution strategies for a pointing task might explain the difficulty of 3D-representation of the performance of participants in other studies as well [30].

## Suggested solutions for an improvement of pointing procedure and device

Pragmatically, to accommodate for both strategies reproducing the spatial location of remembered targets, we decided to use two sets of calibration paradigms: (i) with the eyes open (EO set), where the participant would point towards the intersection of their line of sight, their fingertip and the target and (ii) the “laser set” in which a laser pointer visualized the pointing vector of the arm (e.g., its virtual elongation) in world-space allowing for correction.

While some practical problems of the earlier versions of the handheld device were easily fixable, e.g., randomizing the order of targets, adding a text-to-speech module to give out the target locations from the device itself without providing acoustic cues about the environment or replacing the button-based target confirmation with a wireless system, the problem of comparing the subjects’ pointing performance using their self-centered reference frame as a basis and the measured data in world-based reference frame remained.

To fully reconstruct a participant’s egocentric view of the world in a pointing task as closely as possible, one would require a detailed measurement of the visual input, consisting of (i) the 3D position of each fovea at any given time for the binocular retinal input, (ii) the 3D head tilt for the stereoscopic parallax effect, and (iii) the joint, muscle and soft tissue position of the neck, shoulder, upper arm, lower arm, wrist, hand and finger. A variety of techniques using marker-based photometrics [31] or, e.g., infrared (IRED) sensor arrays [32] have been described to take these details into account, resulting in complex and expensive testing setups.

In our current approach, we focused on creating a simple tool for everyday use. One work-around to the detailed measurement of the aforementioned parameters is the removal of relevant degrees of freedom by controlling the participant’s 3D spatial position and restricting unwanted movement. This has been achieved using seat straps [9, 29] or bite bars [33]. The general idea behind this is that by controlling the subject’s position to a minimal degree, the remaining possible changes of, e.g., foveal position or head tilt can only affect the visual input and the resulting gaze or pointing direction “so much”, i.e., a precise enough pointing device will detect deviations, but the overall improvement gained is negligible.

A modified version of the PointingApp was written that uses the built-in accelerometer data in a smartphone from which a 3D pointing vector can be calculated, assuming a stable gravitational force in the *z*-axis [34] without other accelerational forces present when the device is held in a stationary position. The iPhone® used in this study came equipped with a 6-axis accelerometer/gyroscope unit containing digital-output *X*-, *Y*-, and *Z*-axis angular rate sensors with a programmable range of ± 250, ± 500, ± 1000, and ± 2000°/s (Invensense (TDK), CA, USA). The sensor readouts were acquired, averaged and normalized multiple times after user confirmation. To rule out relevant accelerational forces present which would hamper the vector calculation, a measurement was only recorded when the normalized values after user confirmation did not exceed a ± 5% margin. Otherwise, device motion was assumed and another set of readouts was acquired. From these vectors, a representation in a spherical coordinate system can be derived, giving an azimuth and a polar value. These spherical coordinates could then be compared to the two sets of calibrations. The introduction of spherical coordinates on a unit sphere initially detaches the real-world targets from the performed task, e.g., the subjective direction. By restricting the degrees of freedom in which the subject as the center of the unit sphere can move, the deviation from calibration vector and reproduction vector still yields a meaningful result.

The modified standardized pointing paradigm is as follows: A 3 × 3 matrix is marked with red 20 mm points on a white wall. The central point is marked with a 10 cm wide cross. The distance between points is 100 cm. A viewing angle of 55° between the outer points is achieved by having the participant sit on a revolving chair 192 cm away from the wall. The participant’s eye level can be adjusted by changing the height of the chair and matching it to the central horizontal row of the calibration points. In our clinically oriented setup, we refrain from using bite bars or multi-point straps but strictly control the chair position by adjusting its center of rotation to a defined point marked on the ground. No visual cues are visible on the wall except for a 35 cm wide white power outlet. Only artificial lighting is used. The chair provides only back support but no head or arm rest to minimize proprioceptive input from neck muscles or head position. The only source of sound in the test room is the handheld device itself.

For each task, a computerized voice from the device gives a randomized command, e.g., “top left” in either German or English. The subject points towards the target with an extended arm and confirms the measurement with a wireless Bluetooth dongle with the other hand; alternatively the examiner can confirm the target.

**Fig. 3:**
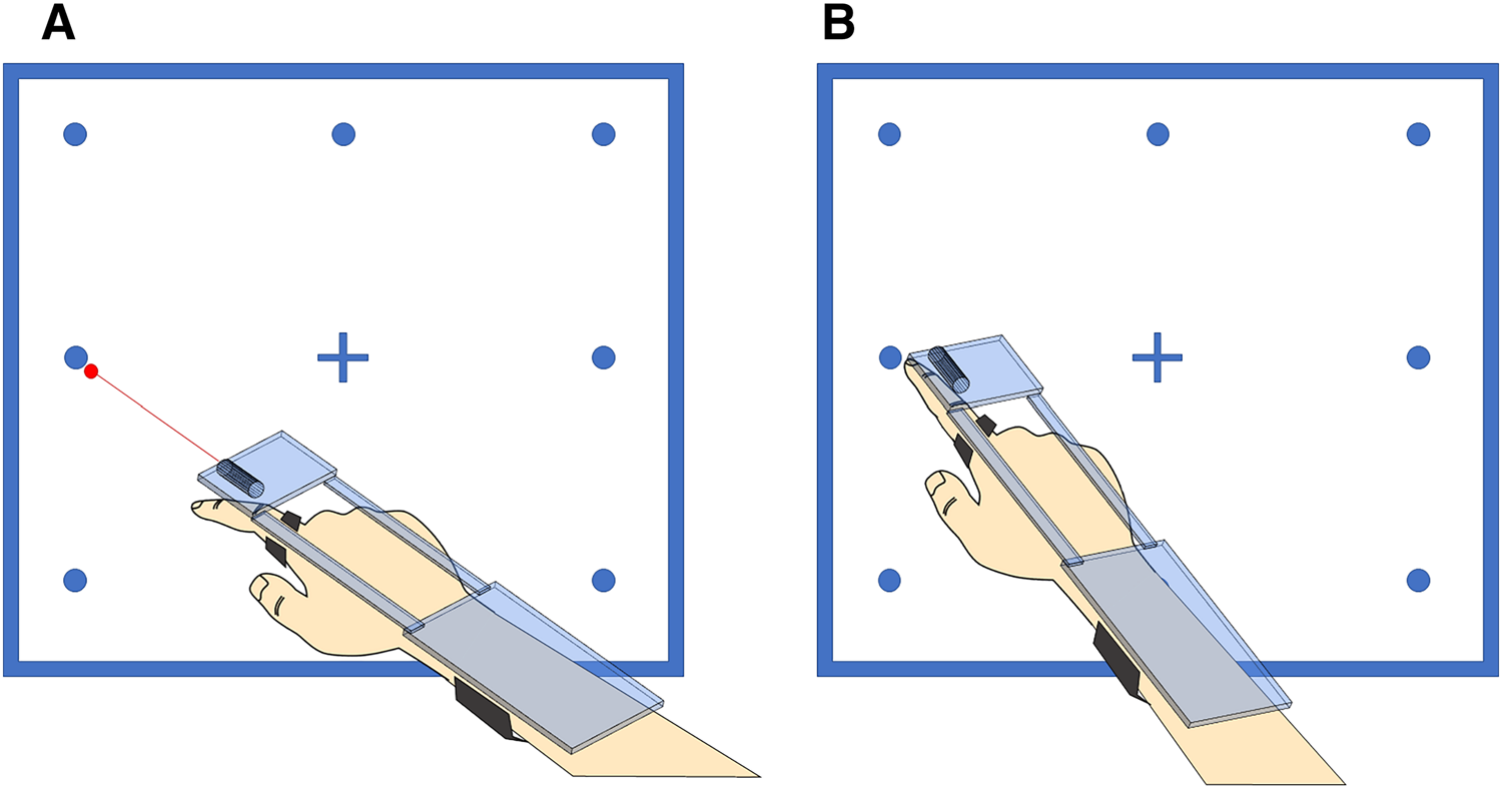
3D-Visualisation from the subjects’ point-of-view, showing the two different calibration paradigms and the optimised pointing device. **A** depicts the laser-based calibration for the target “centre, left” while in **B** no visual aid is provided, requiring the subject to apply a retinotopic strategy to point at the same target instead

Test runs had shown a significant room for error when participants used their wrist mobility performing slight adjustments of their hand position when using a visual aid of their pointing vector (e.g., laser pointer). The synergy of hand, finger and wrist position in precision world-interaction is well known [35]. We, therefore, restricted wrist movement using a three-point attachment system for the device with a forearm strap, a wrist band and a study frame reaching the index finger, where the device is secured with another finger-strap (Fig. 3). To minimize the offset from the laser pointer to the index finger (and its imagined vector) in the world-based calibration, the pointer was moved as close to the pointing finger as possible. For left-handed subjects, the laser pointer could easily be moved on the device. To avoid a subtle change in the center of mass of the frame, the pointer remained in its position even when not needed. The whole frame including the smartphone (Apple iPhone® 6 s, 143 g) weighed 238 g.

We used two different calibration paradigms with (1) eyes open and the attached laser pointer providing feedback on the target and (2) eyes open with the laser pointer switched off, therefore, requiring visual aiming. The target order was randomized in each trial and the chair position remained the same between measurements. The only command given was to “point with a straight arm”. Even in the event of obvious pointing mistakes, the subject was not notified in order not to artificially skew the data (e.g., a patient confusing left and right in the calibrations is likely to also struggle with this distinction in the actual test). If the subjects themselves noticed the error, a repeated measurement of the relevant target was possible. In a pilot method study (see below), a total of ten sets of these calibrations was acquired in five healthy young adults in two consecutive sessions to assess the test–retest-reliability of the method. The data protection clearance and Institutional Review Board of the Ludwig-Maximilians-Universität München, Germany, approved the study (no. 094-10) and all participants gave informed consent. The study was performed in accordance with the ethical standards laid down in the 1964 Declaration of Helsinki and its later amendments. In a future clinical study, measurements of target localization will be performed without visual feedback (closed eyes) and in different body positions after defined active or passive, clockwise or counterclockwise rotations to one side.

## Results of a pilot pointing experiment in healthy volunteers

**Fig. 4:**
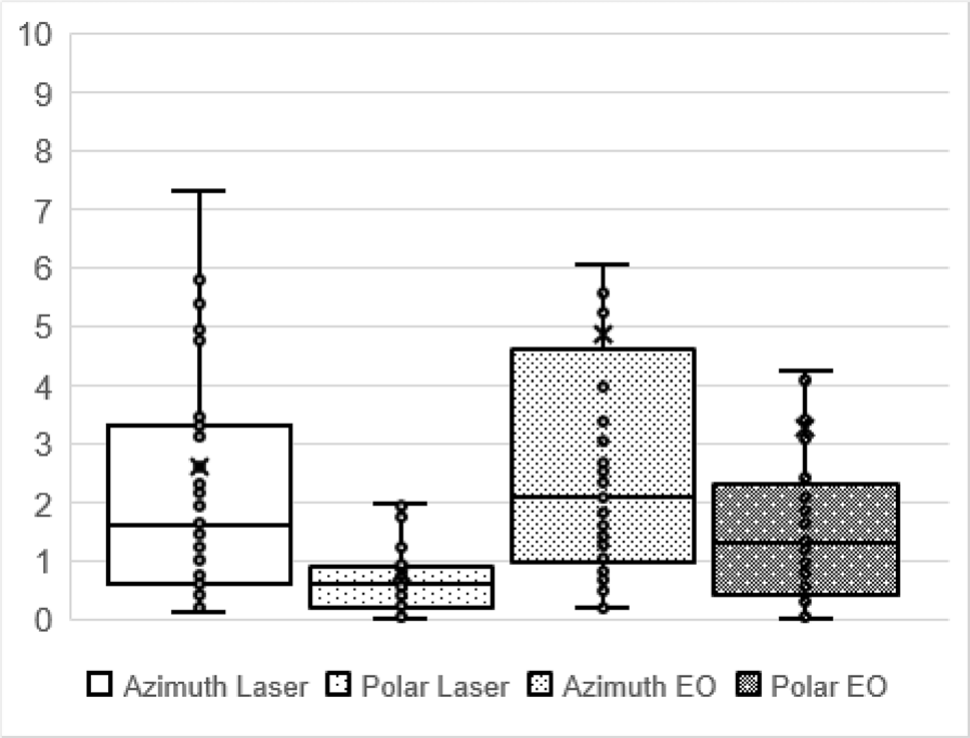
Absolute in-session deviation in degrees (°) from two repeated pointing tests by five healthy volunteers with nine different randomized targets when using laser calibration and eyes open (EO) calibration, respectively. The mean absolute deviation did not exceed 5°, showing a good angular discrimination of the pointing device

Applying the above described standardized testing condition in five participants, the overall test–retest reliability (measured as the mean absolute spherical deviation in degrees for the azimuth and polar planes in two consecutive measurements) was 4.8° (± 10.1°) in the azimuth and 3.3° (± 7.3°) in the polar plane for the eye-based calibration and 2.6° (± 4.3°) in the azimuth and 0.8° (± 1.0°) in the polar plane for the laser-controlled calibration (Fig. 4). Notably, this includes errors due to, for instance, mishearing a voice command and, therefore, pointing towards a wrong target. Excluding obvious errors in a post-hoc analysis gave the following mean deviations: 2.8° (± 4.5°) in the azimuth and 1.6° (± 2.3°) in the polar plane (eye-based calibration) and 2.1° (± 2.9°) in the azimuth plane in the laser-controlled calibration; there were no obvious pointing errors in the polar plane. Even including calibration errors, the mean absolute deviation did not exceed 5° in both the azimuth and the polar plane in healthy participants; our proposed pointing task with an angular difference of 27.5° between targets should, therefore, be resolvable.

Repeating the measurement after 1 week showed a mean deviation between those two tests of 8.6° (± 9.7°) in the azimuth and 5.6° (± 7.9°) in the polar plane in the eye-based calibration and 5.0° (± 4.1°) in the azimuth and 1.9° (± 3.1°) in the polar plane in the laser-controlled calibration.

## Conclusions

Targeted interaction with the world by optimized eye–hand coordination is an everyday task which played a major role in human evolution based on multiple connections between different sensory input networks and motor programs using egocentric or allocentric spatial reference frames.

Spatial orientation and navigation integrate multimodal and sensorimotor input to create a mental map of the surrounding environment. Various explanations of how this mental representation might work on a cellular as well as a systematic level have been proposed. Because of this complexity, testing a patient’s spatial orientation is not simple. Since in neurodegenerative diseases and sensory neurological disorders the underlying pathologies are not easily distinguishable by simple history taking and clinical examination, precise testing methods (easily applicable bedside devices) are required.

As a feasible test for spatial orientation skills of such patients, pointing towards the location of spatial targets provides an elegant and easy to perform bedside test as it does not require expensive machinery and is a common real-life task that involves orientation. Here, we postulate that pointing as a subject–world interaction can be solved by both retinotopic and world-based strategies. Therefore, we modified both our handheld-pointing device and the testing procedure so that the final pointing vector can be evaluated for both strategies. By restricting the individual subject’s movement in 3D space and using a defined spatial position for the tested individuals, we limited the anatomically possible solutions for pointing towards a target, which reached a reasonable test–retest-reliability in two consecutive measurements. Even when repeated 1 week later, the test data showed only minor deviations from the prior performance. A unique calibration is required to define a center for the spherical coordinate system on which the mathematical analysis is based. It shows that even with changes in the position of the device on the subject’s arm and without strictly controlling head, trunk or foveal 3D position, the resulting deviations are small enough for a clinical bedside test.

Our subject group consisting of young and healthy participants showed a high test–retest-reliability and less overall deviation in the laser-based calibration which provides visual world-based performance feedback compared to the visual aiming calibration which can be performed in subjective reference systems, too. We will now begin measurements in neurological patients with cognitive deficits or bilateral vestibulopathy.

## References

[1] Brandt T, Zwergal A, Glasauer S (2017). 3-D spatial memory and navigation: functions and disorders. Curr Opin Neurol.

[2] Kozhevnikov M, Hegarty M (2001). A dissociation between object manipulation spatial ability and spatial orientation ability. Mem Cognit.

[3] Schöberl F, Pradhan C, Grosch M (2021). Bilateral vestibulopathy causes selective deficits in recombining novel routes in real space. Sci Rep.

[4] Schöberl F, Zwergal A, Brandt T (2020). Testing navigation in real space: contributions to understanding the physiology and pathology of human navigation control. Front Neural Circuits.

[5] Bantry White E, Montgomery P (2015). Dementia, walking outdoors and getting lost: incidence, risk factors and consequences from dementia-related police missing-person reports. Aging Ment Health.

[6] Flanagin VL, Fisher P, Olcay B (2019). A bedside application-based assessment of spatial orientation and memory: approaches and lessons learned. J Neurol.

[7] Blohm G, Crawford JD (2007). Computations for geometrically accurate visually guided reaching in 3-D space. J Vis.

[8] Klier EM, Wang H, Crawford JD (2001). The superior colliculus encodes gaze commands in retinal coordinates. Nat Neurosci.

[9] Domkin D, Laczko J, Djupsjöbacka M (2005). Joint angle variability in 3D bimanual pointing: uncontrolled manifold analysis. Exp Brain Res.

[10] Whitwell RL, Ganel T, Byrne CM (2015). Real-time vision, tactile cues, and visual form agnosia: removing haptic feedback from a "natural" grasping task induces pantomime-like grasps. Front Hum Neurosci.

[11] Keefe BD, Watt SJ (2017). Viewing geometry determines the contribution of binocular vision to the online control of grasping. Exp Brain Res.

[12] Proulx MJ, Todorov OS, Taylor Aiken A (2016). Where am I? Who am I? The relation between spatial cognition, social cognition and individual differences in the built environment. Front Psychol.

[13] Kuehn E, Chen X, Geise P (2018). Social targets improve body-based and environment-based strategies during spatial navigation. Exp Brain Res.

[14] Newcombe NS (2018). Individual variation in human navigation. Curr Biol.

[15] Mishkin M, Lewis ME, Ungerleider LG (1982). Equivalence of parieto-preoccipital subareas for visuospatial ability in monkeys. Behav Brain Res.

[16] Goodale MA, Milner A (1992). Separate visual pathways for perception and action. Trends Neurosci.

[17] Crawford JD, Henriques DYP, Medendorp WP (2011). Three-dimensional transformations for goal-directed action. Annu Rev Neurosci.

[18] Karnath H-O, Rüter J, Mandler A (2009). The anatomy of object recognition–visual form agnosia caused by medial occipitotemporal stroke. J Neurosci.

[19] Standage D, Blohm G, Dorris MC (2014). On the neural implementation of the speed-accuracy trade-off. Front Neurosci.

[20] Schöberl F, Pradhan C, Irving S (2020). Real-space navigation testing differentiates between amyloid-positive and -negative aMCI. Neurology.

[21] Levine TF, Allison SL, Stojanovic M (2020). Spatial navigation ability predicts progression of dementia symptomatology. Alzheimers Dement.

[22] Harris MA, Wiener JM, Wolbers T (2012). Aging specifically impairs switching to an allocentric navigational strategy. Front Aging Neurosci.

[23] Sajad A, Sadeh M, Crawford JD (2020). Spatiotemporal transformations for gaze control. Physiol Rep.

[24] Büttner-Ennever JA, Baxter D, Olszewski J et al. (eds) (2014) Olszewski and Baxter's cytoarchitecture of the human brainstem: 1 table, 3. rev. and extended ed. Karger, Basel, Freiburg

[25] Comoli E, Das Neves Favaro P, Vautrelle N (2012). Segregated anatomical input to sub-regions of the rodent superior colliculus associated with approach and defense. Front Neuroanat.

[26] Billington J, Wilkie RM, Field DT (2011). Neural processing of imminent collision in humans. Proc Biol Sci.

[27] Chen X, DeAngelis GC, Angelaki DE (2013). Diverse spatial reference frames of vestibular signals in parietal cortex. Neuron.

[28] Ertl M, EulenburgWoller PM (2021). The role of delta and theta oscillations during ego-motion in healthy adult volunteers. Exp Brain Res.

[29] Henriques DYP, Crawford JD (2002). Role of eye, head, and shoulder geometry in the planning of accurate arm movements. J Neurophysiol.

[30] Vuong J, Fitzgibbon AW, Glennerster A (2019). No single, stable 3D representation can explain pointing biases in a spatial updating task. Sci Rep.

[31] Bruno N, Uccelli S, Viviani E (2016). Both vision-for-perception and vision-for-action follow Weber's law at small object sizes, but violate it at larger sizes. Neuropsychologia.

[32] Henriques DYP, Medendorp WP, Gielen CCAM (2003). Geometric computations underlying eye-hand coordination: orientations of the two eyes and the head. Exp Brain Res.

[33] Burke MR, Grieve KL (2005). Touch responses made to remembered and visual target locations in the dark: a human psychophysical study. Exp Brain Res.

[34] Kok M, Hol JD, Schön TB (2017). Using inertial sensors for position and orientation estimation. FNT Signal Process.

[35] Mason CR, Gomez JE, Ebner TJ (2001). Hand synergies during reach-to-grasp. J Neurophysiol.

